# Epidemiological aspects of suicide attempts among Moroccan children

**DOI:** 10.11604/pamj.2016.24.112.7805

**Published:** 2016-06-02

**Authors:** Nour Mekaoui, Lamiae Karboubi, Fatima Zahra Ouadghiri, Badr Sououd Benjelloun Dakhama

**Affiliations:** 1 Pediatric Medical Emergencies, Children's hospital of Rabat, Morocco; 2 Faculty of Medicine and Pharmacy of Rabat, Mohamed V University Morocco, Morocco

**Keywords:** Suicidal attempts, children, epidemiology

## Abstract

Suicidal behavior among children has significantly increased in Morocco. We conducted a study on the epidemiological aspect to propose a treatment strategy. Descriptive retrospective study over a period of 3 years (April 2012-April 2015) involving children who visited pediatric medical emergencies of the Children Hospital of Rabat after an autolysis attempt. We observed epidemiological parameters, history, social and family context, the means used, the presumed cause, clinical manifestation, and the management. 66 patients were identified. A female predominance was found (sex =15). The average age was 13 years old. This was a first episode in 97% of cases. Psychiatric history was found in 6 patients. The causes of suicide attempt were unidentified in 65%. The most frequent cause was family conflict (35%). The most frequent method was pharmaceutical drug ingestion (54.4%). Children were asymptomatic (57.6%). Neurological manifestations (30%) were most frequent. Digestive symptoms (12%) and hemodynamic (3%) were also discovered. Patients were hospitalized in a general pediatric service 92.4% of the times, admitted to intensive care 4.5% of the times, and two patients refused to be hospitalized. The treatment consisted of gastric lavage (18%) supplemented by symptomatic measures. The outcome was favorable in 95.4% of cases. We recorded 2 deaths by rat poison poisoning. All patients were advised in writing after leaving to follow up with a psychological treatment. Suicide attempts are the result of an ill being, mostly among children living in a family with conflict. Upstream treatment is essential to identify children at risk. Additionally, a psychiatric care in hospital is essential to avoid recurrences.

## Introduction

Suicide attempts (SA) are a curse and a result of children living in distress. In Morocco, suicidal behavior in children and youth are clearly increasing [1]. In France and The United States, they represent respectively the second and the third cause of mortality worldwide [2]. We studied the epidemiological aspects and the various circumstances that led to the attempt to autolysis to identify risk factors and the aspects of the treatment of these children in order to provide an appropriate response strategy adapted to our context.

## Methods

This is a descriptive retrospective study conducted over a period of 3 years (April 1, 2012 to April 1, 2015) involving all children who visited pediatric medical emergencies of Children's Hospital of Rabat after an attempt to autolysis. Children included were at the age of 16 or under whose suicide attempt context was identified: children confession or strong suspicion by the surroundings and caregivers. Children with a strong suspicion of domestic violence or accident were excluded. The epidemiological parameters (age, sex, socio-economic, and cultural level); personal history: history of suicide attempt, known psychological disorder, chronic disease; family and social context: family conflict, parents divorce, adoption, the means used and the presumed cause, clinical data (clinical state admission), scalable and received treatment were recorded, standardized, then and data were collected and analyzed using excel. Qualitative data were expressed as percentages, the quantitative data in medians (minimum - Maximum) and average standard deviations.

## Results

**Figure 1:**
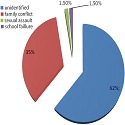
Presumed causes of suicide attempts

**Figure 2:**
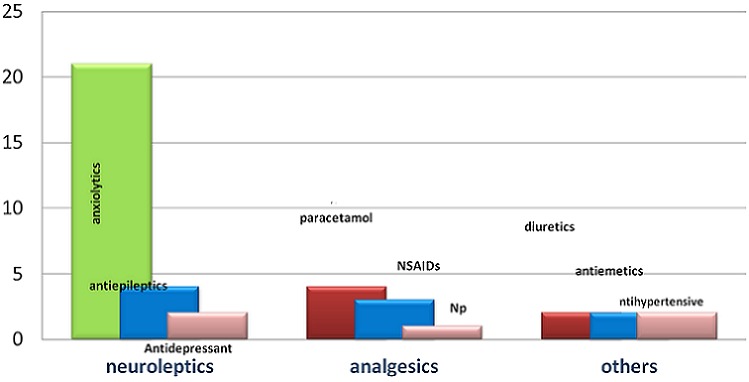
Distribution of drugs used for suicide attempt

Sixty six patients were identified. Predominance among female was found with a sex ratio of 15 (93.75% female, 625% male). The average age was 13 years old (11-16 years). This was a first episode in 97% of cases. Psychological history was found in 6 patients with a psychosis and 2 cases with depression symptoms. The presumed causes of suicide attempt were not found in 65% of cases. The most frequent cause was family conflict with 35% of cases. Other causes such as school failure and sexual assault have also been found in 1.5% of the cases (Figure 1). The most frequent methods used were consumption of pharmaceutical drug with 54.4%, and with a predominance of anxiolytics used 58% of the times and followed by analgesics with 17% of the times (Figure 2). Poorly drug intoxication was found in 25% of cases and included over 10 tablets in 90% of cases. A rat poison or organophosphate product was used 34.4% of the times. Among other means used were caustic ingestion (1 case), one case of cannabis intoxication and a case of strangulation in a child with psychosis treatment. Most of the children were asymptomatic (57.6%). Clinical manifestations were dominated by neurological manifestations (30%) essentially consciousness disorders (90%) of which 22.2% were comatose on admission. Other signs were found, including dizziness and one case of aphasia. Digestive symptoms (12%) as nausea, vomiting and abdominal pain have also been found. 3% of patients had hemodynamic disorders at admission (Table 1). These were cases of PPD and organophosphates poisoning. Patients were mostly hospitalized in a general pediatric unit (92.4%) and intensive care for severe cases (4.5%). Two patients refused hospitalization. The therapeutic treatment consisted of gastric lavage in 18% of cases associated with symptomatic measures. The outcome was favorable in 95.4% of cases. We recorded 2 deaths, both due to rat poison poisoning. The children died a few minutes after admission. All our patients were referred after leaving with a letter to follow up with a psychological treatment.

**Table 1:** Clinical manifestations at admission

Clinical manifestations	%
Asymptomatic	57.6%
**Neurological:**	**30%**
Coma	22%
Obnubilation /somnolence	6%
Vertigo	1%
Aphasia	1%
**Digestive:**	**12%**
Nausea	10%
Vomitting	8%
Abdominal pain	4%
**Hemodynamic trouble**	**3%**

## Discussion

Suicide in children is a taboo subject and is still poorly understood. In our Arab Muslim context, it remains a difficult subject to approach and is perceived as a “disgrace” by family and society. At present, suicide attempts represent a public health problem in industrialized countries because of their increasing frequency and its potential severity. A study of Tournemire R. et al. 2010, conducted in young children of 12 to 18 years showed that 8.3% of girls and 6.9% of boys have already made a suicide attempt [3, 4]. In Morocco, the occurrence of suicide attempt is unknown. We identified 60 cases over a period of 3 years; this low rate suggests an underestimation of suicide attempt rates in the general population. In fact, only patients whose context was obvious have been included: child or family's confession or cases of mass poisoning in a meaningful context. Epidemiological studies in the general population could bring a significant contribution in the occurrence of suicide attempts in children and youths in Morocco. Knowledge of risk factors or predictors is essential to give to these children an appropriate preventive care.

According to epidemiological studies, several factors have been identified: Age: suicide attempts more often involve teenagers [5]. They are rare in people under 13 years and often reveal an underlying psychological disorder. They represent 10-15% of SA among children and youth [3, 6]. In our study, children under 13 years accounted for 4.5% of all SA. Gender: we noted female predominance in both age groups (under 12, and more than 12 years). However this proportion increases with age respectively to reach 33% and 94%. This dominance is largely found in the literature [7] and is explained by the fact that girls have more severe suicidal ideation (23.6%) during the critical period of pre-adolescence [8]. Underlying psychiatric illness: would promote the transition to the act and up in relapse. Known psychiatric history was found in 4 patients with recurrence in 50% of cases. The presence of a psychiatric disorder multiplied by a factor of 11 to 27 the risk of SA over the general population. A history of TS seems to increase by 25% the risk of recurrence [9]. The few antecedents found in our study does not exclude a psychiatric disorder (depression, psychosis) previously unrecognized.

The environmental and social context: family conflicts were the most frequent cause. They were essentially conflicts with one parent, reflecting a stress factor, probably without real desire of death. Although it means a call for help, or a resumption of the dialogue, the passage to the act should not be neglected, and should raise attention and adequate monitoring. In literature, other factors have been identified such as maltreatment, sexual abuse and neglect [10, 11]. These cases were found in 3 patients, however; they must be researched systematically before being reported. Poverty is a factor of vulnerability of the occurrence of a suicide attempt [11]. In our study, most patients were of low socioeconomic class. Harmful pharmaceutical drugs are the most used by girls. The boys, however, used more phlebotomy, and violent means [8]. The use of drug ingestion was found in work and for people from different cultures: Tunisia [9], France [12] and Congo [13]. This modality was, in our work, specific to girls, which is also consistent with the literature [14]. Our treatment consisted of hospitalization associated to symptomatic treatment. Patients were thereafter sent with a letter to child psychiatry consultation. Many shortcomings were identified in our care including lack of psychological care in the first 24 hours and the absence of the medical system established enabling support for the three aspects: medical, psychological, and social.

We propose a strategy to support a child in the context of suicide attempt in the emergency: Reception and evaluation of 3 components: somatic, psychological and social; in the emergencies: the first contact with the health care team must be in a climate of empathy, trust and confidentiality. Assessment of vital functions, looking for signs of abuse, including sexual abuse, conditioning, gastric lavage if necessary, symptomatic treatment, if the child has a good conscience: start psychological assessment within 24 hours by a psychiatrist. The objectives of the initial interview is the collection of the first psychic complaints, the study of the context of the crisis, and the search for a possible psychiatric pathology and severity of signs that may involve risk of recurrence in the short term including maltreatment and sexual abuse. The interview with parents or relatives is essential. He is rising the own experiences, collect their difficulties and complaints, and appreciate the quality of life outside the hospital. The social assessment must identify the social context of the entourage, school or employment status of the child, his level of adaptation and possible existence of a current social monitoring. After emergency stage, hospitalization is required where the psychological, family and social evaluation must be pursued in parallel to the initiation of the physical care.

## Conclusion

Suicide attempts are a manifestation of ill-being in children and occur most often in a situation of family conflicts. Preventive treatment is essential to identify children at risk to commit a suicide attempt. The involvement of various people in contact with the child is essential to identify children at risk (teachers, school psychologists, social workers) and address them for follow up. A psychiatric care in hospital is essential for all children who attempted suicide to avoid recurrences.

### What is known about this topic

- The suicidal attempt is going more frequent but still underestimated.
- It's occurrence in adolescent.
- Therapeutic measures include physical and psychological support.

### What this study adds

- Description of the underestimation of suicidal behavior in Arab Muslim context.
- Illustration of the management difficulties in an African country.
- A proposal of management strategy in a developing country
